# Production of Carbon Occluded in Phytolith Is Season-Dependent in a Bamboo Forest in Subtropical China

**DOI:** 10.1371/journal.pone.0106843

**Published:** 2014-09-04

**Authors:** Zhang-Ting Huang, Pei-Kun Jiang, Scott Xiaochuan Chang, Yan Zhang, Yu-Qi Ying

**Affiliations:** 1 School of Environment and Resources, Zhejiang A&F University, Lin’an, Zhejiang, China; 2 Zhejiang Provincial Key Laboratory of Carbon Cycling in Forest Ecosystems and Carbon Sequestration, Zhejiang A&F University, Lin’an, Zhejiang, China; 3 Department of Renewable Resources, University of Alberta, Edmonton, Alberta, Canada; University of Missouri, United States of America

## Abstract

Carbon (C) occluded in phytolith (PhytOC) is a stable form of C; when PhytOC is returned to the soil through litterfall it is stored in the soil which can be an effective way for long-term C sequestration. However, few estimates on the rate of PhytOC input to the soil are available. To better understand the seasonal dynamics of PhytOC production and the annual rate of stable C sequestration through PhytOC input, we quantified the monthly litterfall, phytolith and PhytOC return to the soil over a year in a typical Lei bamboo (*Phyllostachys praecox*) forest in subtropical China. The monthly litterfall ranged between 14.81 and 131.18 g m^−2^, and the phytolith concentration in the monthly litterfall samples ranged between 47.21 and 101.68 g kg^−1^ of litter mass, with the PhytOC concentration in the phytolith ranged between 29.4 and 44.9 g kg^−1^ of phytolith, equivalent to 1.8–3.6 g kg^−1^ of PhytOC in the litterfall (based on litterfall dry mass). The amount of phytolith input to the soil system was 292.21±69.12 (mean±SD) kg ha^−1^ yr^−1^, sequestering 41.45±9.32 kg CO_2_−e ha^−1^ yr^−1^ of C in the studied Lei bamboo forest. This rate of C sequestration through the formation of PhytOC found in this study falls within the range of rates for other grass-type species reported in the literature. We conclude that return of C occluded in phytolith to the soil can be a substantial source of stable soil C and finding means to increase PhytOC storage in the soil should be able to play a significant role in mitigating the rapidly increasing atmospheric CO_2_ concentration.

## Introduction

Phytolith, or plantstones or plant opal, is a noncrystalline form of mineral (SiO_2_) that deposits in the intra- and extra-cellular structures of different plant tissues after the absorption of soluble silica by plant roots in the form of monosilicic acid (Si(OH)_4_) [1]–[3]. Phytolith is essentially a silicified feature that forms as a result of biomineralization that occurs within plants and during the process some organic C can become occluded in the phytolith (PhytOC) during its formation in plant tissues [2], [4]. Phytolith typically is highly resistant to decomposition [5], [6], being stable in both aerobic and anaerobic conditions and in soils with pH ranging between 3.5 and 9.8 [7], [8]. As such, the PhytOC is much more stable than organic C fractions in the soil that are not occluded in phytolith [2], [9]. When plant litter decomposes after it falls onto the soil, PhytOC as a stable form of C will accumulate with phytolith and remain in the soil for a long period of time [2]. The accumulation of PhytOC in the soil is an important millennium scale mechanism for long-term C sequestration in terrestrial ecosystems, which could potentially contribute 15–37% of the estimated global soil organic C (SOC) sequestration of 24 kg C ha^−1^ yr^−1^; this form of C sequestration could play a positive role in mitigating global climate change [2], [5], [10], [11].

Despite the potential importance of phytolith in SOC stabilization and long-term C storage, few estimates exist on the concentration of phytolith in different plant species and ecosystem types [12]–[14]. Measurements of phytolith and PhytOC concentrations in different ecosystem types are urgently needed to improve our understanding of the potential contribution of PhytOC in global C sequestration [15]. Such data would also help us design management practices, such as the selection of species or genotypes that have high phytolith concentrations, to take advantage of this mechanism for SOC sequestration to reduce atmospheric CO_2_ concentration [16].

Past research indicates that plants in the Poaceae family, a large and nearly ubiquitous family consisting of monocotyledonous flowering plants, have relatively high phytolith concentrations [3], [17]. Among the more than 10,000 species in the Poaceae family, bamboo species have particularly high phytolith concentrations in their tissues [3]. Plants in the Poaceae family thus have an advantage in sequestering organic C in the form of PhytOC accumulation in the soil [2], [5], [11], [12], [15], [18]. Through the determination of phytolith and PhytOC concentrations in different bamboo species, combined with the published bamboo litterfall rate [19], [20], Parr et al. [15] estimated the organic C sequestration potential through PhytOC accumulation in bamboo forests of ten economically important bamboo species. They concluded that the global contribution to C sequestration on an annual basis is equivalent to 11% of the anthropogenic CO_2_ release [15]. The work of Parr et al. [15] and others [16] illustrated the advantage of bamboo forests in long-term C sequestration through the formation of PhytOC. However, one potential deficiency with the work of Par et al. [15] is that the PhytOC contribution was estimated by its content in bamboo leaves rather than through their existence in the litterfall or in the soil. In reality, only PhytOC returned to the soil through litterfall and accumulated in the soil can be considered a contribution to SOC sequestration. The most reliable estimate of the rate of PhytOC input is through quantifying litterfall rate and the PhytOC concentration in the litterfall [16]. Since PhytOC return in litterfall constitutes PhytOC return to the soil, the seasonal dynamics of PhytOC return in litterfall should be understood but that has not been studied.

In this study, we determined the annual rate of PhytOC input to the soil in a Lei bamboo forest in subtropical China through measuring the amount of litterfall and the PhytOC concentration in the litterfall to provide an estimate of the potential contribution of C sequestration in the form of PhytOC input to the soil, as well as the seasonal dynamics of PhytOC input to the soil. Lei bamboo belongs to the Bambusoideae subfamily in the Poaceae family and is a species with high economic value due to the production of bamboo shoot, a widely used delicacy in Southeast Asia. This species is widely planted in southern China and the area planted to this bamboo species has increased dramatically in the last several decades [21]. As such, studying the rate of PhytOC production in Lei bamboo forests can improve our understanding of the potential contribution of this form of C sequestration in Lei bamboo forests and should have implications for estimating PhytOC input to the soil in forests of other bamboo species. The objectives of this study were therefore to determine the amount of litterfall and the associated PhytOC input to a Lei bamboo forest on a seasonal basis to understand the dynamics of both litterfall and PhytOC input through monthly sampling of the studied bamboo forest and to understand the contribution of PhytOC input from other bamboo components and obtain ecosystem level PhytOC storage and compare that with PhytOC storage in other ecosystems reported in the literature. Specifically, we hypothesize that 1) PhytOC production through litterfall in bamboo forests has a strong seasonal pattern and 2) the annual PhytOC return to the soil is a substantial contribution to soil C sequestration particularly as compared to rates of soil C sequestration in different ecosystems reported in the literature.

## Materials and Methods

### Ethics statement

No specific permits were required for the described field studies involving the collection of litterfall samples from the studied bamboo forest. The bamboo forest used in our sampling was privately owned and was not protected in any way and thus a specific permit for not-for-profit research is not required. The field studies did not involve endangered or protected plant or animal species in the study area.

### Study area

The study site was located in Congkeng Village in Shankou Township (30°14′N, 119°42′E), Lin’an City, Zhejiang Province, in southeast China. The elevation of the site was 150 m above sea level and the general local landform was dominated by low hills. The study area had a monsoonal subtropical climate with four distinct seasons with hot and humid summers and cold winters. The average annual temperature and average annual precipitation of the study site were16°C and 1414 mm, respectively, based on the climate data between 2000 and 2009, with most of the precipitation occur between April and October. The site had an average of 236 frost-free days and 1946 day-light hours. The soils of the experimental area were classified as Anthrosols in the FAO soil classification system [22].

The site selected for this study had the bamboo forest established 10 years ago, with Lei bamboo density at 2,045 stems ha^−1^ and a diameter at breast height (1.3 m from the ground) of 3.9 cm. Those bamboo forests were managed for bamboo shoot production, a form of bamboo forest management that yields a high economic return [21]. The studied bamboo forest was converted from rice paddy in 2001, 10 years before this study was initiated in 2011. The bamboo forest was intensively managed as described in Li et al. [21].

### Experimental design and sampling

Four randomly selected sampling sites were established in the bamboo forest described earlier. One 10×10 m plot was set up within each site and within each plot we set up four 1×1 m litter traps. Litter samples were collected on a monthly basis between January and December 2011. At each litter sample collection, the sample in each litter trap was transferred to a plastic bag, brought back to the laboratory, weighed, dried and weighed again. Water content was determined from those weights. The dried samples were ground and stored for chemical analysis. The main objective of this part of the study was to understand the seasonal dynamics of PhytOC return to the soil through bamboo litter fall.

In the second part of this research, we determined the biomass of standing bamboo in the bamboo stand we studied and compared that to the bamboo leaf litter production determined in the first part of the research described above. The main objective was to understand the partition of PhytOC in different bamboo components and compare that with bamboo leaf litter. This would also allow us to compare the ecosystem level PhytOC content with other ecosystems reported in the literature. In this part of the research, four sites were selected in 2012 in the studied stand after the new bamboo shoots produced in the spring were fully grown to a bamboo plant in late June. In each site, one 10×10 m plot was established. Within each plot, all bamboo plants were surveyed, with their average diameter and density, and grouped based on their age (all bamboo plants were from one to four years of age). After the field measurement was complete, three bamboo plants of each of the four ages representing a diameter gradient were harvested to determine the bamboo leaf biomass for each of those bamboo plants. The bamboo leaf samples from the three bamboo plants of each age were then mixed and 1 kg of each of those samples was collected and brought back to the laboratory. Once in the laboratory, the bamboo leaf samples were washed with deionized water, dried at 105°C for about an hour to quickly remove the majority of the water, and the samples were then dried at 65°C to constant weight to determine the water content of the leaf samples. The samples were ground and the phytolith and PhytOC concentrations determined using the methods described below.

The C and nitrogen (N) concentrations in the litter samples were determined based on a dry combustion method using an Elementar Vario MAX CN analyzer (Elementar Analysensysteme GmbH, Hanau, Germany). A microwave digestion method was used in this study to isolate phytolith from the litter samples [23]. After the microwave digestion, the samples were treated with a Walkley-Black type digestion [24] to ensure that extraneous organic materials in the samples were completely removed in recovering the phytolith sample [15]. The isolated phytolith samples were oven-dried at 75°C for 24 h in a centrifuge tube of known weight and the quantity of phytolith recovered was determined. The C concentration in the phytolith samples was then analysed on a Thermo Finnigan Flash EA 1112 CHNS-O Analyser (Thermo Finnigan, San Jose, CA). A soil standard sample (GBW07405) and a plant standard sample (GBW07602) were included in the analysis as part of the quality control measures. Precisions of better than 5% in the measurement of phytolith and better than 8% in the measurement of C concentration in phytolith were achieved through analysis of duplicate samples.

### Data analysis

The phytolith concentration in litterfall samples, the C concentration in the phytolith, and the PhytOC concentration in litterfall samples were calculated using the following formulas:
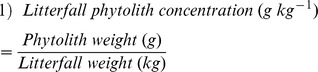


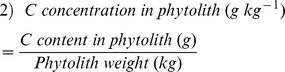


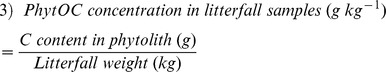



The PhytOC addition rate from litterfall was calculated by multiplying the litterfall weight, the phytolith concentration and the C concentration in phytolith or by multiplying the litterfall weight and the PhytOC concentration in litterfall samples.

The normality and homogeneity of variance were tested before performing the ANOVA analysis and data were log-transformed if the assumptions for ANOVA were not met. An one-way analysis of variance (ANOVA) was conducted to test the effect of sampling month on the amount of litterfall, SOC concentration in the litterfall, and phytolith concentration, C concentration in phytolith and the PhytOC concentration in the litterfall. When the ANOVA analysis indicated a significant treatment effect, the Duncan’s new multiple range test (MRT) was used to separate the means. Correlation analysis was performed to evaluate relationships between the studied parameters. An alpha level of 0.05 for significance was used in all statistical analyses, unless mentioned otherwise. All of the statistical analyses were performed using the SPSS software (SPSS 18.0 for windows, SPSS Inc., Chicago, USA).

## Results

**Figure 1:**
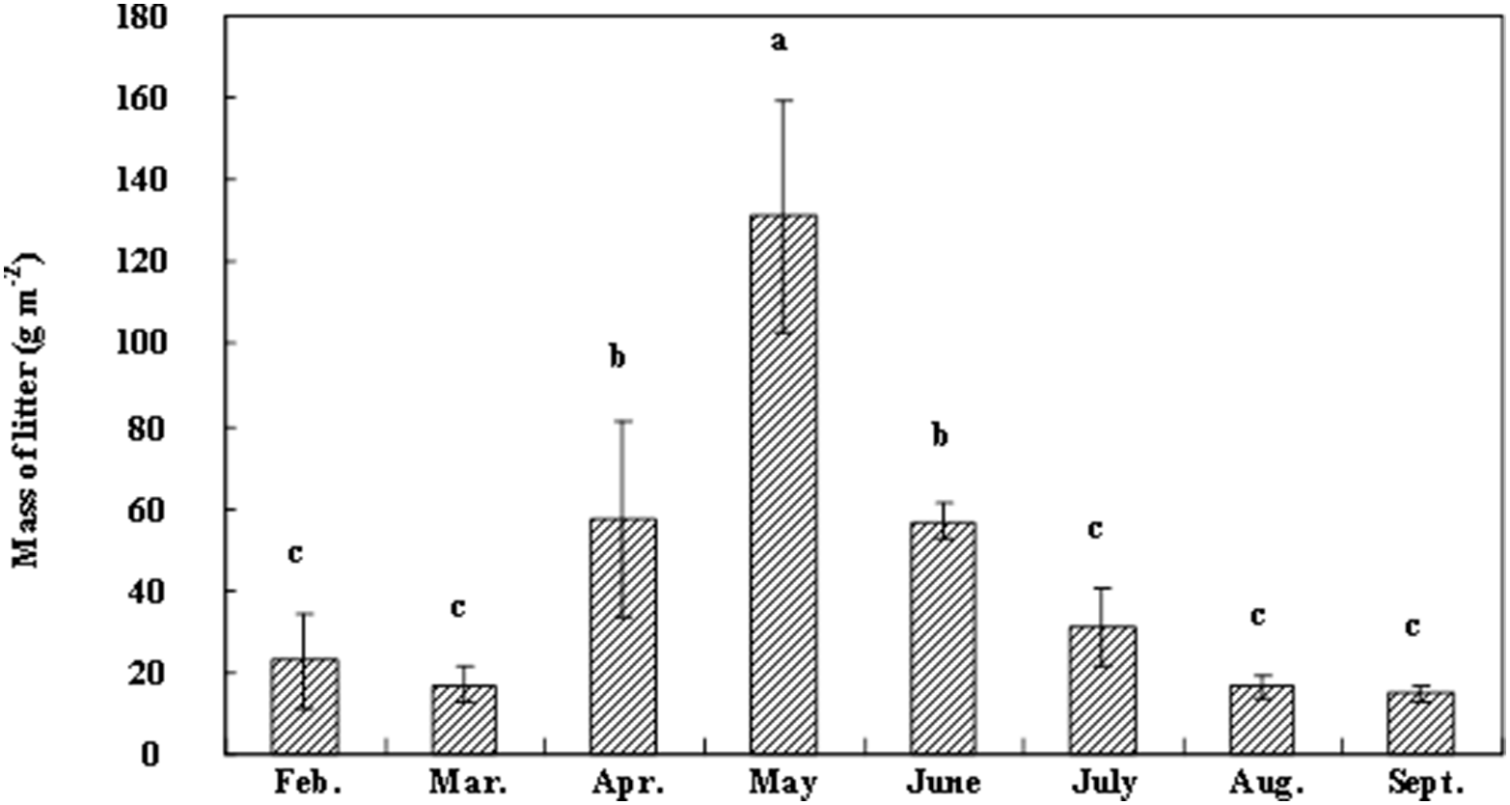
Seasonal dynamics of litter fall mass (oven-dry basis) in a Lei bamboo forest in subtropical China. Vertical error bars are standard deviations. Means followed by different lowercase letters are not significantly different.

The amount of litterfall collected in each month was quite variable, with litterfall rates ranging from 14.81 to 131.18 g m^−2^. The rates of litterfall were greater between April and June than in other months of the year, with the greatest litterfall rate obtained in May (Fig. 1). No litterfall was collected between October and January. The total amount of litterfall in the study year was 347.61 g m^−2^.

The C concentration in the litterfall samples did not change dramatically between the sampling months but tended to be higher in late fall than in spring or summer (Table 1). The SiO_2_ concentration in the litterfall samples markedly changed with the month of litterfall sample collection, with the highest concentrations found in late winter/early spring and the lowest in the summer growing season (Table 1). The seasonal change in the PhytOC concentration in the phytolith was not consistent with the seasonal change of the phytolith concentration in the litter samples, with a non-significant negative correlation between the two (R^2^ = 0.11, P>0.05; data not shown). When the PhytOC contained in the litter sample was expressed on the basis of the litter biomass (Table 1), the PhytOC concentration was positively correlated with the phytolith concentration (R^2^ = 0.51, P<0.01; data not shown).

**Table 1:** Carbon, SiO_2_, phytolith and PhytOC concentrations (or stocks), and PhytOC fluxes through litter fall in a Lei bamboo stand in different months.

Months	Litter biomass(g m^−2^)	C concentration(g kg^−1^)	SiO_2_ concentration(g kg^−1^)	Phytolith concentration(g kg^−1^)	PhytOC inphytolith (g kg^−1^)	PhytOC inlitter (g kg^−1^)	Phytolith stock(kg ha^−1^ yr^−1^)	PhytOC stock(kg ha^−1^ yr^−1^)	PhytOC fluxes(kg CO_2_–e ha^−1^ yr^−1^)
February	22.95±11.56 c	457.27±5.53 bc	76.55±8.91 bc	77.16±7.48 c	29.4±9.1 b	2.2±0.6 c	18.21±10.11 cd	0.51±0.32 c	1.86±1.19 c
March	17.00±4.17 c	451.76±2.16 cd	88.74±5.13 ab	88.09±3.42 bc	35.9±11.5 ab	3.2±1.1 ab	14.99±3.75 cd	0.52±0.15 c	1.90±0.55 c
April	57.25±23.81 b	442.13±3.32 e	91.93±6.98 a	101.68±6.99 a	34.6±4.3 ab	3.5±0.5 a	58.00±23.92 b	1.95±0.67 b	7.15±2.45 b
May	131.18±28.24 a	444.43±7.51 de	83.29±14.51 ab	90.37±15.03 ab	40.4±3.2 a	3.6±0.6 a	121.59±48.45 a	4.89±1.88 a	17.94±6.88 a
June	56.89±4.45 b	446.98±11.41 de	67.96±2.75 c	80.78±3.28 bc	43.6±2.4 a	3.5±0.2 a	45.88±2.47 bc	2.00±0.15 b	7.33±0.54 b
July	31.01±9.64 c	463.03±5.53 b	39.24±11.07 d	53.61±7.49 d	44.9±3.8 a	2.4±0.5 bc	17.00±7.06 cd	0.77±0.36 c	2.81±1.31 c
August	16.52±2.72 c	476.42±4.34 a	45.69±2.44 d	47.21±3.81 d	38.7±8.0 ab	1.8±0.2 c	7.86±1.75 d	0.30±0.05 c	1.09±0.17 c
September	14.81±2.29 c	472.52±3.49 a	47.93±4.70 d	58.68±7.89 d	43.9±5.7 a	2.5±0.2 bc	8.70±1.88 d	0.38±0.06 c	1.38±0.21 c
Total/Average	347.61±41.16	/	/	/	38.9±1.4	2.9±0.2	292.21±69.12	11.30±2.54	41.45±9.32

Values are means and SD. Means followed by the same lowercase letters in a column are not different among the month of litter collection.

The monthly amount of phytolith and PhytOC returned to the soil through litterfall (Table 1) was highly correlated (R^2^ = 0.98, P<0.01; data not shown). The total amount of PhytOC returned to the soil through litterfall was 11.30 kg ha^−1^ yr^−1^, the equivalent of removing 41 kg of CO_2_ ha^−1^ yr^−1^.

The total bamboo green leaf biomass in the studied bamboo stand was 3.79 ton ha^−1^ (Table 2), which was slightly greater than the 3.48 ton ha^−1^ of the annual bamboo leaf litter determined (Table 1). The phytolith contained in the green bamboo leaf was only 101.86 kg ha^−1^ (Table 2), far less than the 292.21 kg ha^−1^ that can be returned to the soil through litterfall (Table 1). Consistent with the phytolith content in the standing bamboo leaves, the amount of PhytOC that was contained in the green bamboo leaf was also substantially less than that contained in the bamboo leaf litter.

**Table 2:** Biomass, phytolith and PhytOC concentrations and content (or stock), and PhytOC fluxes in fresh leaves in a Lei bamboo stand.

Age ofleaf (yr)	Biomass(t ha^−1^ yr^−1^)	PhytolithConcentration(g kg^−1^)	PhytOC inphytolith(g kg^−1^)	Phytolith stock(kg ha^−1^ yr^−1^)	PhytOC stock(kg ha^−1^ yr^−1^)	PhytOC fluxes(kg CO_2_−e ha^−1^ yr^−1^)
1	0.06±0.02 c	9.91±3.10 d	60.03±1.7 a	0.54±0.07 b	0.03±0.003 c	0.12±0.01 c
2	2.09±0.63 a	21.58±3.95 c	56.03±5.2 a	44.53±13.43 a	2.46±0.59 a	9.03±2.18 a
3	0.94±0.51 b	31.29±5.68 b	57.30±17.5 a	27.46±10.64 a	1.49±0.40 b	5.45±1.48 b
4	0.70±0.29 bc	41.33±8.38 a	51.18±4.4 a	29.32±13.82 a	1.54±0.82 b	5.64±3.02 b
Total	3.79±1.01	/	/	101.86±22.67	5.52±1.36	20.24±4.99

Values are means and SD. Means followed by same lowercase letters in a column are not different among the ages of leaves.

## Discussion

With the continued increase of greenhouse gas concentrations in the atmosphere and the related increase of the climatic forcing [25], the need to enhance C sequestration through biological means to mitigate the increasing greenhouse gas concentrations is becoming more urgent [26]. One of the main ways to biologically increase C sequestration is to increase C storage in the soil. The soil stores a vast amount of organic C and has the capacity to store more. However, organic C storage in the soil has different residence times and some of the SOC is very vulnerable to loss through mineralization under the actions of microbial populations in the soil [27], [28]. Mechanisms for long-term SOC preservation in terrestrial ecosystems include physical protection of chemically recalcitrant organic matter within organo-mineral complexes [29], formation of charcoal [30], and organic C occlusion in phytolith [2]. The last mechanism has recently been promoted as a potential means to increase the long-term storage of SOC as PhytOC is considered a highly stable C that can be stored in the soil for hundreds of years [13]–[16].

The strong seasonal variation in PhytOC concentration in litterfall samples (Table 1) in the studied bamboo forest supported our first hypothesis. Such seasonal variations are to be expected as the litterfall may be consisted of leaves formed in different years and PhytOC concentrations in leaves change with leaf age [31]–[33]. Our own work also supports the literature data that PhytOC concentrations are dependent on the age of the leaf (Table 2). The implication of this is that we need to collect litter samples throughout the year to obtain accurate information about PhytOC production in bamboo forest ecosystems.

This is one of the first papers that report PhytOC concentrations in litter samples and the contribution of litterfall to the return of PhytOC to the soil [16]. Most earlier determinations on the contribution of plant species to ecosystem C sequestration through PhytOC production was based on estimates of PhytOC concentrations in green leaf samples [9], [13], [14], [34]. This paper demonstrates that the input of PhytOC to the soil through litterfall is much greater than estimates derived from leaf biomass and PhytOC concentrations in green leaf (Tables 1 and 2) [15]. Our data indicate that the PhytOC input to the soil estimated using the method in Parr et al. [15] using phytolith concentrations in green leaves can be quite different from PhytOC input measurements based on leaf litter input and PhytOC concentrations in leaf litters. Those differences are likely caused by the different sampling times of the plant samples used for analysis of PhytOC concentrations, as phytolith concentrations increase with plant age [31]–[33]; as a result of that, the PhytOC concentrations in the leaf litter (the oldest leaves on a plant) should be higher than that in the green leaves, as was demonstrated in this study (Tables 1 and 2). Therefore, determining the amount of litterfall and the PhytOC concentration in the litterfall is a more accurate method to quantifying the PhytOC return to the soil in bamboo forest ecosystems.

The large difference in the PhytOC concentration between the bamboo leaf and bamboo leaf litter resulted in the two-time difference in the amount of C that can be sequestered in the form of PhytOC that were estimated through the two methods. This indicates that the amount of C sequestration in the form of PhytOC in previous publications (e.g., [15]) that were based on PhytOC concentrations in green leaves would represent an underestimate. If we follow the same approach used in Parr et al. [15] and consider that the global potential land that can be cultivated (4.1×10^9^ ha) is planted to bamboo or plant species with similar PhytOC production potential, the global potential to sequester atmospheric CO_2_ is going to be greater than the 11% of the current global CO_2_ emissions suggested by Parr et al. [15].

We reviewed the literature and summarized the silicon and phytolith concentrations, biomass production, and the C sequestration potentials (expressed as CO_2_ equivalent) of various plant species known to have high abilities to form phytolith (Table 3). The literature data indicate that silicon concentrations in plant biomass are variable, with the highest silicon concentrations reported in sugarcane plants. Silicon concentrations in wheat, bamboo, rice and some aquatic plants can also be high. The highest PhytOC concentration is also reported in sugarcane, followed by wheat, bamboo and rice. The 11.30 kg ha^−1^ yr^−1^ of PhytOC returned to the soil through litterfall (equivalent to the removal of 41 kg of CO_2_ ha^−1^ yr^−1^) supported our second hypothesis that the amount of annual PhytOC return to the soil is a substantial contribution to soil C sequestration. The C sequestration potential through PhytOC formation of the bamboo forest we studied falls within the range reported in other grass-type species [11]–[15], [18]. The literature data indicate that the C sequestration potential through PhytOC formation was high in some aquatic plant species [14]. Despite that the PhytOC concentrations in different plant species in the Poaceae family are not dramatically different, the PhytOC production potential can be quite different largely because of the large differences in biomass production (Table 3).

**Table 3:** PhytOC fluxes associated with different plant species based on data from the literature.

Plant species	PhytOC inphytolith (g kg^−1^)	PhytOC inbiomass (g kg^−1^)	Biomass(t ha^−1^ yr^−1^)	PhytOC fluxes(t CO_2_−e ha^−1^ yr^−1^)	Reference
Lei bamboo(litter)	38.9±1.4	2.9±0.2	3.48±0.41	0.042±0.009	This study
Sugarcane	38.8∼192.6	0.9∼2.5	40	0.123∼0.364	[13]
Economicbamboo species	16.0∼40.2	2.4∼5.2	1∼37	0.008∼0.709	[12]
Foxtail millet	25.1±12.7	1.4±0.7	4.09	0.020±0.010	[8]
Common millet	19.2±14.3	1.3±0.9	4.79	0.023±0.015	[8]
Wheat	13.0∼129.0	0.6∼6.0	2.10∼11.20	0.006∼0.246	[14]
Rice	14.2∼33.6	0.4∼2.8	9.30∼18.60	0.030∼0.130	[24]
Riparian plants	4.9∼39.7	0.1∼1.6	0.01∼11.50	0.052∼23.562	[25]
Shallow-wateremergent plants	11.0∼23.3	0.1∼1.5	16.00∼34.06	3.775∼159.359	[25]
Floating-leafaquatic plants	9.1∼19.4	0.2∼0.4	0.46∼99.18	0.320∼87.278	[25]

Values represent the range or mean±SD.

For most economically important bamboo species, only bamboo leaf litter will be returned to the soil while bamboo stem, which would constitute the majority of biomass production in a bamboo forest, would be exported out of the ecosystem as an economic product that can be used for making furniture or other products. Eventually, the PhytOC contained in those bamboo parts would be returned to the broader ecosystem and still contribute to C removal from the atmosphere [35]. Therefore, by only assessing the PhytOC content in the soil [36] and the contribution of PhytOC through litterfall [15] will substantially underestimate the potential for C sequestration in bamboo forests. Full accounting of the PhytOC sequestration potential of bamboo forests at the ecosystem level will be required to fully understand their C sequestration potential.

In summary, the production of litterfall and the concentration of phytOC in the litterfall should be used to calculate the potentials of bamboo forests for stable C sequestration in the form PhytOC. The PhytOC concentration in litterfall had seasonal variations and thus representative seasonal litter samples should be collected for analysis to provide more accurate estimates of the potential for PhytOC formation and climate change mitigation. PhytOC production can make a substantial contribution to long-term C sequestration in the studied bamboo forest. In estimating the contribution of PhytOC production for C sequestration in bamboo forests, PhytOC removal in the form of bamboo products from the ecosystem should also be considered.
